# Expanding the Genetic Landscape of Craniofacial Anomalies Through Transcriptome-Wide Association Studies

**DOI:** 10.21203/rs.3.rs-7645057/v1

**Published:** 2025-10-17

**Authors:** Elly Brokamp, Alexandra Scalici, Tyne Miller-Fleming, David Wu, Wendy K. Chung, Monica H. Wojcik, Nancy J. Cox, Megan M. Shuey

**Affiliations:** Vanderbilt University Medical Center; Vanderbilt University Medical Center; Vanderbilt University Medical Center; Vanderbilt University Medical Center; Boston Children’s Hospital; Boston Children’s Hospital; Vanderbilt University Medical Center; Vanderbilt University Medical Center

**Keywords:** Craniofacial anomalies, transcriptome-wide association studies, congenital anomalies, electronic health records

## Abstract

**Background:**

Craniofacial anomalies are common congenital anomalies that significantly contribute to infant mortality and life-long health problems. Studies of craniofacial anomalies have identified several genetic causes, but focus on rare, Mendelian presentations. Despite this, current diagnostic genetic testing only identifies a causal genomic variant in ~ 25% of affected individuals. This low diagnostic yield for Mendelian conditions may relate to oligogenic and polygenic risks for craniofacial anomalies. In this study we sought to use large electronic health record systems including many patients with craniofacial anomalies to determine whether we could identify patterns of genetic associations with craniofacial anomalies and known associated genes.

**Methods:**

We performed transcriptome-wide association studies that evaluated the association between genetically predicted gene expression and craniofacial anomalies in two cohorts: Vanderbilt University Medical Center’s BioVU and Electronic Medical Records and Genomics Network (eMERGE). Using a list of 391 previously identified craniofacial anomaly-associated genes we determined whether there was a greater proportion of significant associations with these genes than others. We also evaluated whether these genes were associated with other congenital anomalies.

**Results:**

We determined the predicted expression of 12 (3.1%) of the known craniofacial anomaly genes were associated with craniofacial anomalies (p < 0.05) in BioVU and 18 (4.6%) in eMERGE. In both cohorts, the majority of significant genes and those demonstrating the strongest significance were not previously associated with craniofacial anomalies. In total, we identified 53 genes not previously associated with craniofacial anomalies. Interestingly fewer than 15% of the known craniofacial associated genes were associated with craniofacial anomalies (p < 0.05) while 262 (76.8%) were associated with congenital anomalies of the heart, 133 (39.0%) anomalies of the nervous system and 142 (41.6%) of the urinary system.

**Conclusions:**

Our results support that both rare and common variation in Mendelian disease-associated genes may contribute to craniofacial anomalies and are broadly involved in congenital anomaly development.

## Background

Craniofacial anomalies (CFAs) are a common group of congenital anomalies (CAs) caused by the abnormal development of skull and/or facial bones. The most common CFAs, cleft lip with or without cleft palate (CL/P) and cleft palate alone, make up one third of all CAs in the United States. They occur in ~ 16 in 10,00 births within the United States and are a major contributor to infant mortality.^1^ Likewise, craniosynostosis, another common CFA, is estimated to affect 1 in 2100–2500 births and can result in abnormal brain growth causing neurological dysfunction.^2^ The frequency, contribution to infant mortality, and life-long associated health problems associated with CFAs makes understanding of genetic underpinnings of the risk and presentations of CFAs essential.

Despite a great deal of research into the genetic etiology of CFAs, clinical genetic testing for CFAs has a relatively low diagnostic yield. There is little understanding of what drives the variable expression and incomplete penetrance of CFA syndromes. Combined diagnostic approaches of karyotype, chromosomal microarray, and exome sequencing can detect the genetic cause of 22.5% of individuals with orofacial clefts.^3,4^ Similarly, comprehensive clinical diagnostic genetic testing of individuals with craniosynostosis can detect a genetic cause in about a quarter (25%) of affected individuals.^5^ These results suggest that, at best, for every four patients with a CFA who access comprehensive genetic testing, one will receive a diagnostic results for the genetic cause of their condition. Even when a diagnostic test identifies a causal variant in a Mendelian gene, there is often incomplete penetrance and/or variable expressivity, suggesting the presence of complex genomic and environmental interactions that impact phenotypic manifestations. As sequencing technologies have advanced and sample sizes have increased, several studies have examined how polygenic variation impacts the penetrance of rare variants associated with CFAs such as cleft lip and palate.^6,7^

To better understand how polygenicity impacts CFAs, large sample sizes are essential. Resources such as Vanderbilt University Medical Center’s BioVU and the Electronic Medical Records and Genomics (eMERGE) network cohort, which link genotype data to electronic health record (EHR) data, could contribute to these efforts.^8,9^ We therefore undertook the first use of these resources to evaluate the genetically predicted changes in gene expression of CFA-associated Mendelian genes as well as the genetically predicted gene expression of individuals with CFAs.

## Methods

### Identification of Individuals with Craniofacial and Other Congenital Anomalies

**Figure 1.**
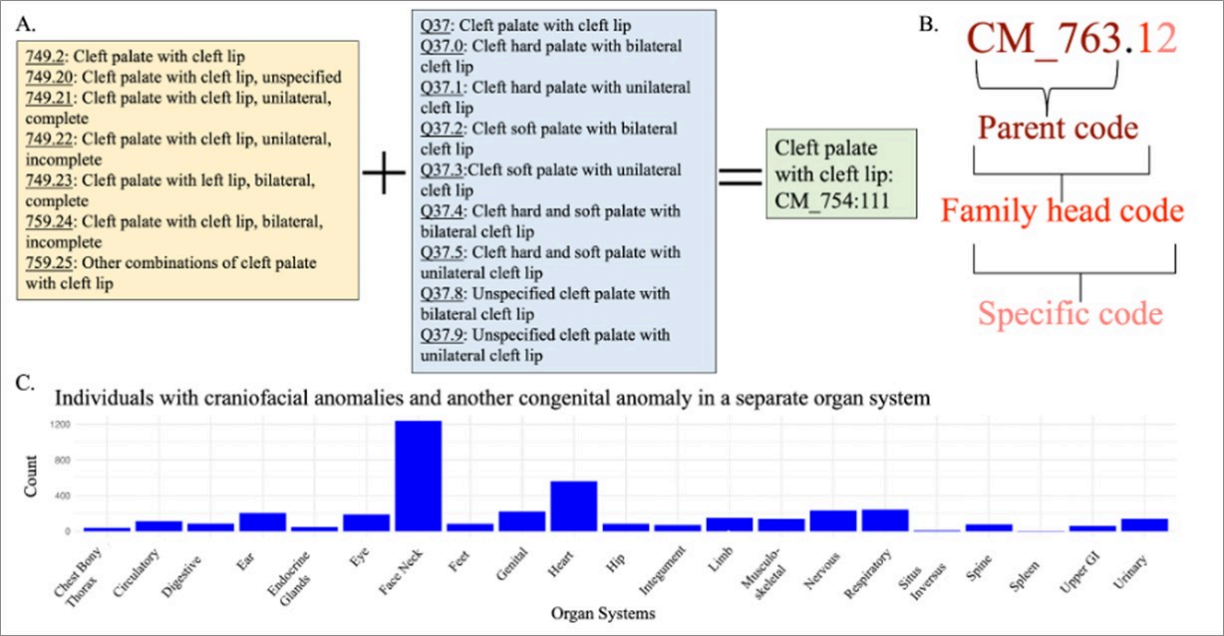
Craniofacial Anomaly Phenotyping. (**A**) Demonstrates phecodeX data structure, e.g how related International Classifiers of Disease (ICD) version 9 and 10 billing codes are collapsed into a single phecode. Seven ICD9 codes and nine ICD10 codes are collapsed into the single cleft palate with cleft lip phecode (CM_754.11). (**B**) Demonstrates the different components of a phecode, parent code, family head code, and the entire specific code. Cases for the TWAS were classified as having two or more instances of a phecode within the same family head code. Cases for the congenital anomaly wide phenotype association were classified as having two or more instances of the same specific code. (**C**) In VUMC 21.1% of individuals with craniofacial anomalies have another congenital anomaly. This bar chart shows the number of individuals with another congenital anomaly on the y-axis and is grouped by the specific organ system of the other congenital anomaly.

We used the phecodeX version of phecodes, systematic groupings of International Classifiers of Disease (ICD) billing codes, which are part of the CM (congenital malformation) chapter of phecodes to identify individuals with CAs.^10^ The Tongue, Mouth, and Pharynx parent code (CM_754) and the Skull, Face, and Jaw parent code (CM_755) within the CM chapter were used to identify individuals with CFAs. Figure 1A demonstrates how ICD9 and ICD10 billing codes are collapsed into one phecode. All available billing codes from an individuals’ entire medical history are used in the construction of phecodes. We mapped the CM phecodes from the ICD code data using the PheWAS R package.^11^

### Identification in Vanderbilt University Medical Center

Vanderbilt University Medical Center’s EHR-system provides a unique resource with a wealth of de-identified health information in the synthetic derivative (SD). The SD contains ~ 3.5 million individuals, with ~ 300k individuals having genetic information in BioVU, VUMC’s deidentified EHR-linked biobank. 6,131 Individuals with CAs and CFAs were identified from Vanderbilt University Medical Center (VUMC)’s de-identified EHR-linked DNA biobank, BioVU.^8,12^ This work is deemed non-human subjects work by the VUMC IRB and received all necessary approvals. We restricted our cases and controls used in analyses to a medical home population (n = 1,275,576), a high medical use population defined as individuals with an ICD-9 or 10 billing code collected from at least three unique visit dates over three or more years. This medical home definition ensures a study population with substantial phenotypic information within their EHR. All genetic data for included participants in BioVU were genotyped using the Illumina MEGAEX Array and the included population was restricted to participants of European genetic ancestry based on clustering in principal component analysis (PCA) using genetic data from the 1000 Genomes Project as reference populations, as previously described.^13^ This restriction was done to minimize confounding results and maximize sample size. Future work to include individuals of non-European genetic ancestry is ongoing.

### Identification in eMERGE

We identified individuals with CFAs from the Electronic Medical Records and Genomics (eMERGE) Network, which combines DNA biorepositories with EHR data.^9,14^ The eMERGE GWAS cohort includes 64,536 individuals from five institutions (VUMC, Columbia University Irving Medical Center, Northwestern Medical Center, Mass General Brigham, and Cincinnati Children’s Hospital Medical Center (CCHMC)). Analyses were restricted to participants of European genetic ancestry from CCHMC in the eMERGE GWAS cohort. Because 75% of CFA cases in eMERGE were from CCHMC we restricted our secondary analysis to this population. ICD billing codes for each individual are provided as part of available data to eMERGE consortium researchers. We mapped the CM phecodes from the ICD code data using the PheWAS R package as previously described.^11^

### Identifying Genes with Known CFA Associations

We curated a list of genes associated with CFAs, using previously published genome-wide association study (GWAS) results and the Concert Genetics’ registry of clinical genetic tests.^15,16^ This testing registry aggregates all clinically available genetic panels, lists all the genes included in each panel, and allows for comparison of included genes between different companies’ panels. These panels reflect a largely comprehensive list of genes with a known monogenic CFA association. A certified genetic counselor searched Concert Genetics’ Test Registry using the search term “craniofacial” and compiled a list of all the unique genes that are offered on the resulting Craniofacial Panel Tests. These panels included both syndromic and non-syndromic CFA genes. Supplemental Table 1 lists curated known CFA-associated genes.

### Defining CFA cases and controls

To identify individuals in BioVU and eMERGE that have a CFA, we used phecodes from the “Tongue Mouth and Pharynx” and the “Skull Face and Jaw” phecodeX Chaps. 1° We identified individuals that had at least two instances of a CFA phecode of the same parent code and/or family head code Supplemental Table 2. Figure 1B demonstrates the breakdown of the parent code, family head code, and specific code portions of a phecode. We defined controls as individuals who did not have a single congenital anomaly phecode in their record.

### Defining CA cases and controls

To define whether individuals in BioVU and eMERGE had CAs we used phecodes from the congenital malformations phecode X Chap. 1^0^ For our cases, we identified individuals with at least two instances of a specific CA phecode (Fig. 1B, Supplemental Table 3). Controls were defined as individuals with no CA phecodes in their record.

### Transcriptome Wide Association Study of Individuals with CFAs

Using machine learning models such as PrediXcan, UTMOST, and Joint Tissue Imputation (JTI), we calculated genetically predicted gene expression (GPGE) using GTEx (version 8) as a reference population in both BioVU and eMERGE.^17,18,19,20^ Using the single best performing model out of these three models for each gene tissue pair (r^2^ > 0.01), we conducted a transcriptome-wide association study (TWAS). We tested the association of CFA status with GPGE in a logistic regression model adjusting for age, sex, number of visits, and the first ten principal components of ancestry.

### Association of Craniofacial Anomaly Genes with Other Congenital Anomalies

To determine how variation in GPGE of known CFA genes may increase risk for other CAs, we conducted gene-based phenotype-wide association studies (PheWAS) of the known CFA genes.^21,22,23^ Of the 391 known CFA genes, we had quality prediction for 341 (r^2^ > 0.1). We tested whether a diagnosis of a CA is associated with GPGE of a known CFA gene adjusting for age, sex, the first ten principal components of ancestry, and number of visits in a logistic regression mode.

## Results

### Identification of individuals with craniofacial and other congenital anomalies within the EHR

Within the entire VUMC medical home population, including individuals without genotype data, there are 19,509 individuals with a CFA. About a quarter of these individuals, 4,051 (21.1%) have a second CA in another organ system (Fig. 1C). At VUMC, there are 694 individuals of European ancestry with a CFA who have available genotype information (248 individuals with a “Tongue, Mouth, and Pharynx” phecode CA and 446 individuals with a “Skull, Face, and Jaw” phecode CA). From the eMERGE GWAS cohort individuals of European ancestry at CCHMC, there are 384 individuals with a CFA (113 individuals with a Tongue, Mouth, and Pharynx phecode CA and 322 individuals with a Skull, Face, and Jaw phecode CA) (Table 1).

### TWAS of individuals with CFAs identifies genes not previously associated with CFAs

**Figure 2.**
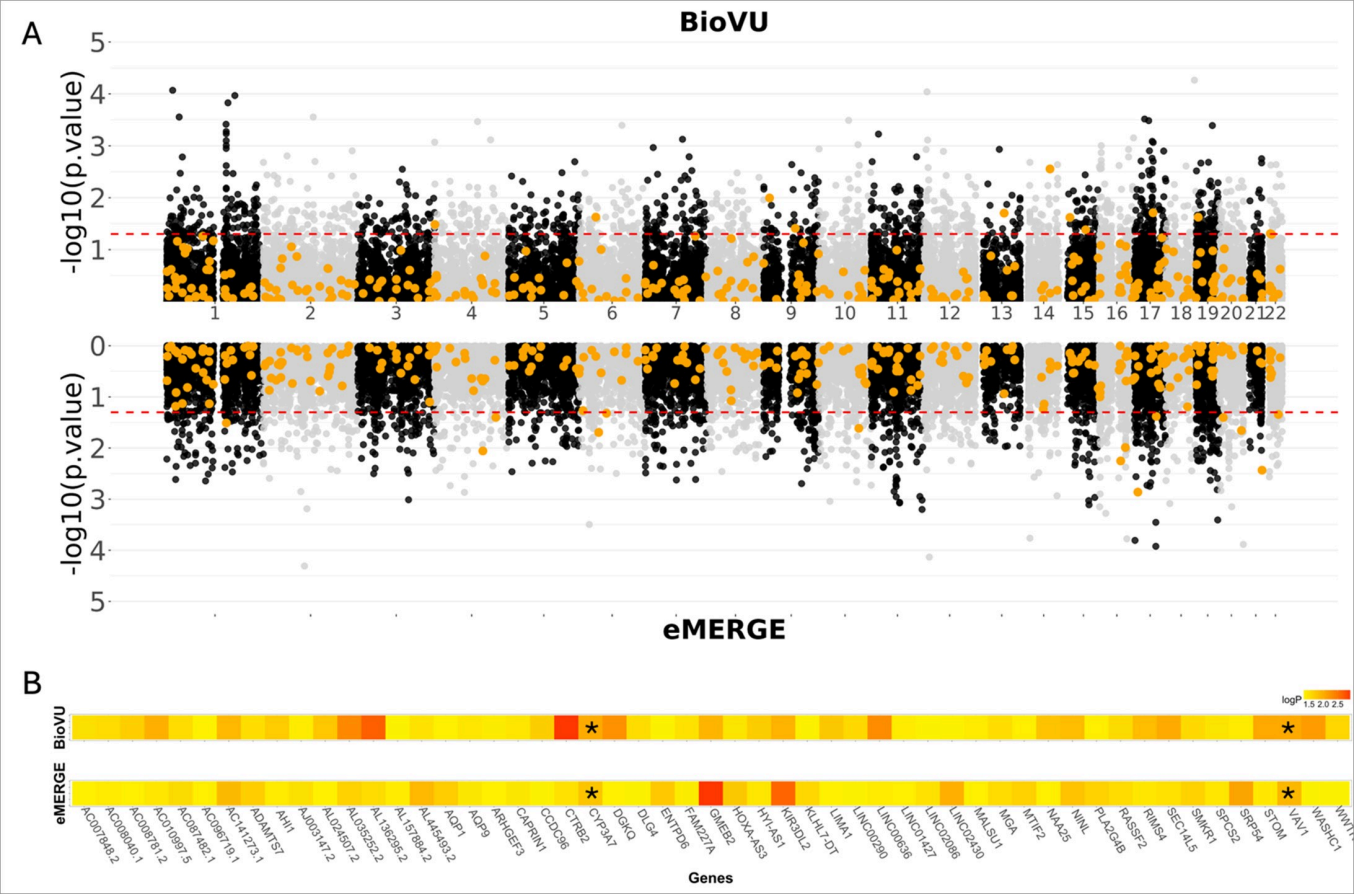
BioVU and eMERGE Craniofacial Anomaly (CFA) Transcriptome wide association study (TWAS) results. TWAS analyses identify genes not previously associated with CFAs. **(A)** Miami style plot of genes with GPGE associated with CFAs in BioVU and eMERGE. The dotted red lines indicate a p-value of 0.05 and the orange points highlight our curated list of known CFA genes. **(B)** A heatmap of the 53 genes that were significant (p<0.05) in both BioVU and eMERGE cohorts. The asterisk (*) indicates the two genes associated with CFAs in both BioVU and eMERGE with a p<0.01.

Our TWAS of CFAs in both BioVU and eMERGE did not identify any statistically significant associations that passed the highly conservative Bonferroni multiple testing correction (0.05 divided by the number of genes with GPGE per tissue). When using a less stringent p-value threshold, we identified 1,261 genes in BioVU and 1,260 gene in eMERGE that were significantly associated (p < 0.05). We compared these genes in both study populations to our curated list of known CFA genes and found that the majority of our curated CFA genes (93.6%) demonstrate no level of significant association, even at a permissive p < 0.05 level, with CFAs in either BioVU and eMERGE (Fig. 2A). In total, fewer than 1% of significant genes in either BioVU or eMERGE were part of the curated previously known CFA-associated gene list. This included 11 significant genes from BioVU (0.90%) and 14 (1%) from eMERGE (Table 2). There was no overlap in significant genes known to association with a CFA in either BioVU or eMERGE.

Additionally, we compared the 1,261 and 1,260 significant associations (p < 0.05) in each study population and found 53 significant gene associations that were shared between the BioVU and eMERGE TWAS results (Fig. 2B). These 53 genes were not part of the curated gene list and were not identified previously as associated with CFAs or craniofacial structure. By using a more stringent p-value threshold (p < 0.001), we identified 231 genes in BioVU and 257 genes in eMERGE associated with CFAs (Table 2). From this more stringent cutoff we identified two genes, *VAV1* (*164875) (BioVU p = 0.009; eMERGE p = 0.006) and *CYP3A7* (*605340) (BioVU p = 0.009; eMERGE p = 0.009) that are shared between both cohorts (Fig. 2B). Neither of these genes were associated with CFAs or any human disease but have been implicated in development.^24,25^

#### CA-wide association study illustrates that known CFA genes are associated with a broad range of CAs across multiple organ systems

**Figure 3.**
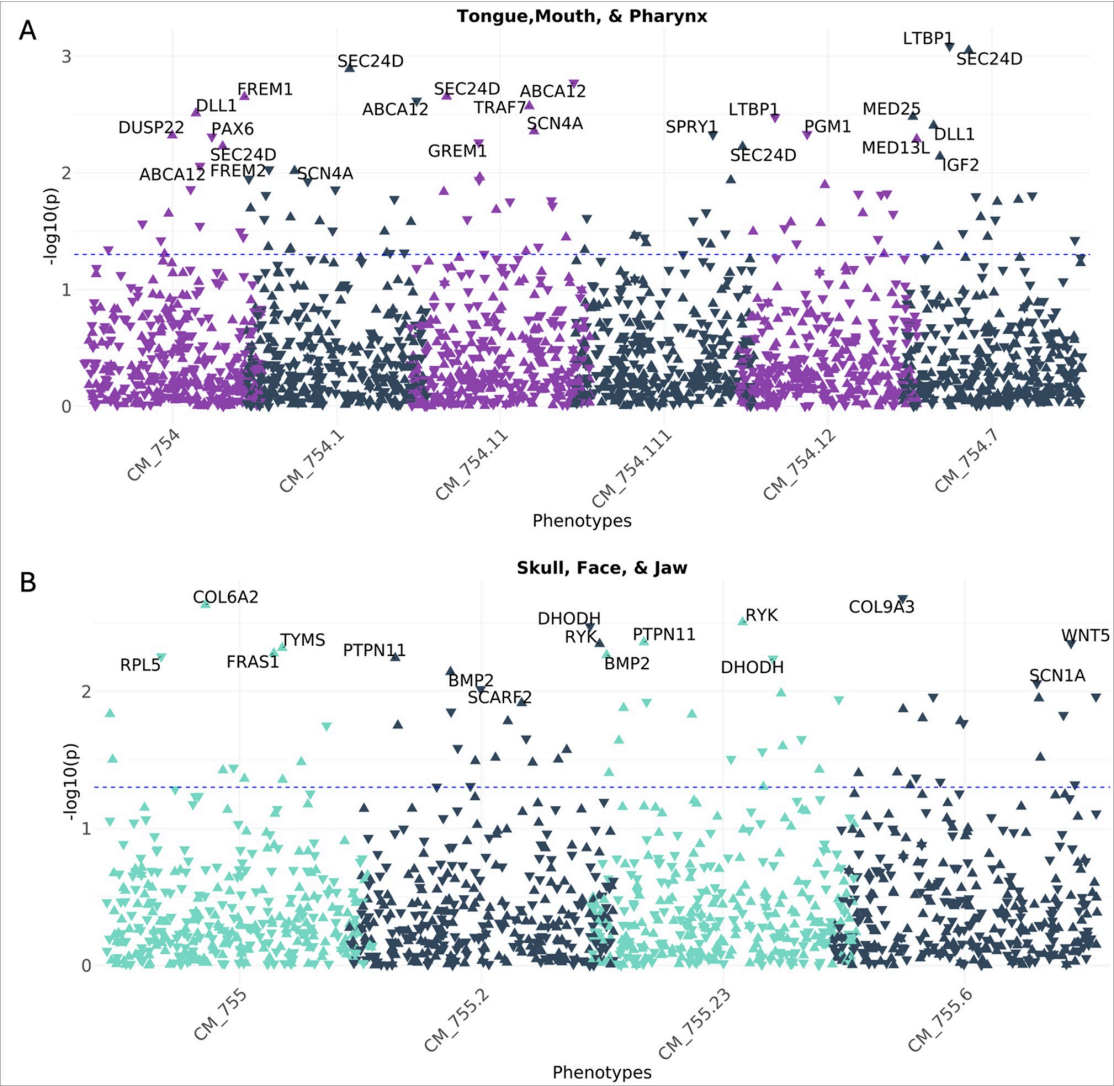
Known Craniofacial Anomaly (CFA) genes associated with CFA phecodes in BioVU. We demonstrate significant associations between known genes and specific phecodes based on the specific phecodeX chapters **(A)** Skull, Face, and Jaw and (**B)** Tongue, Mouth, and Pharynx chapter. The blue dotted line indicates a p-value of 0.05. Direction of the triangle aligns with the direction of the effect.

**Figure 4.**
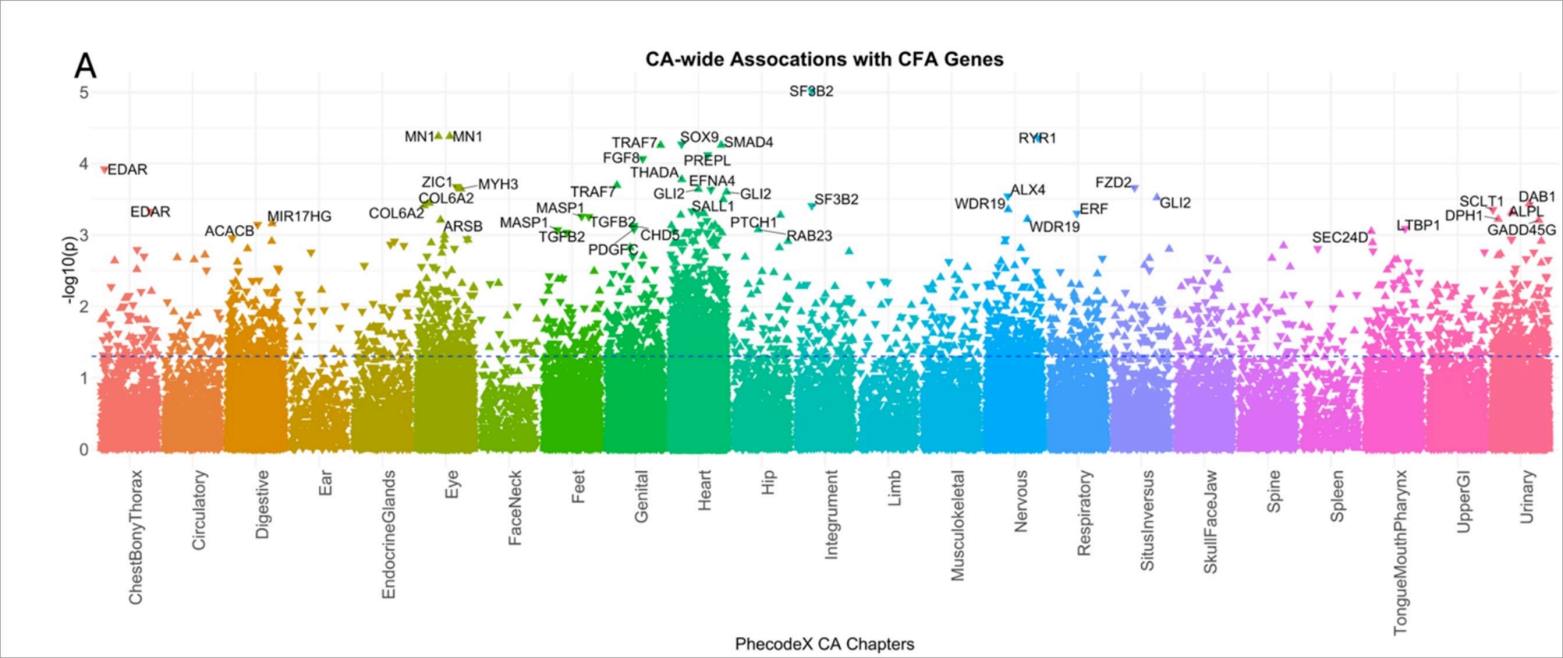
Association study of genetically predicted gene expression of known craniofacial anomaly (CFA) genes and other congenital anomalies (CA). We demonstrate significant associations between known CFA genes and specific phecodes from congenital malformation phecodeX chapters based on organ system. The plot is the known CFA gene associations with other congenital anomalies in BioVU. The blue dotted line indicates a p-value of 0.05. Direction of the triangle aligns with the direction of the effect.

**Figure 5.**
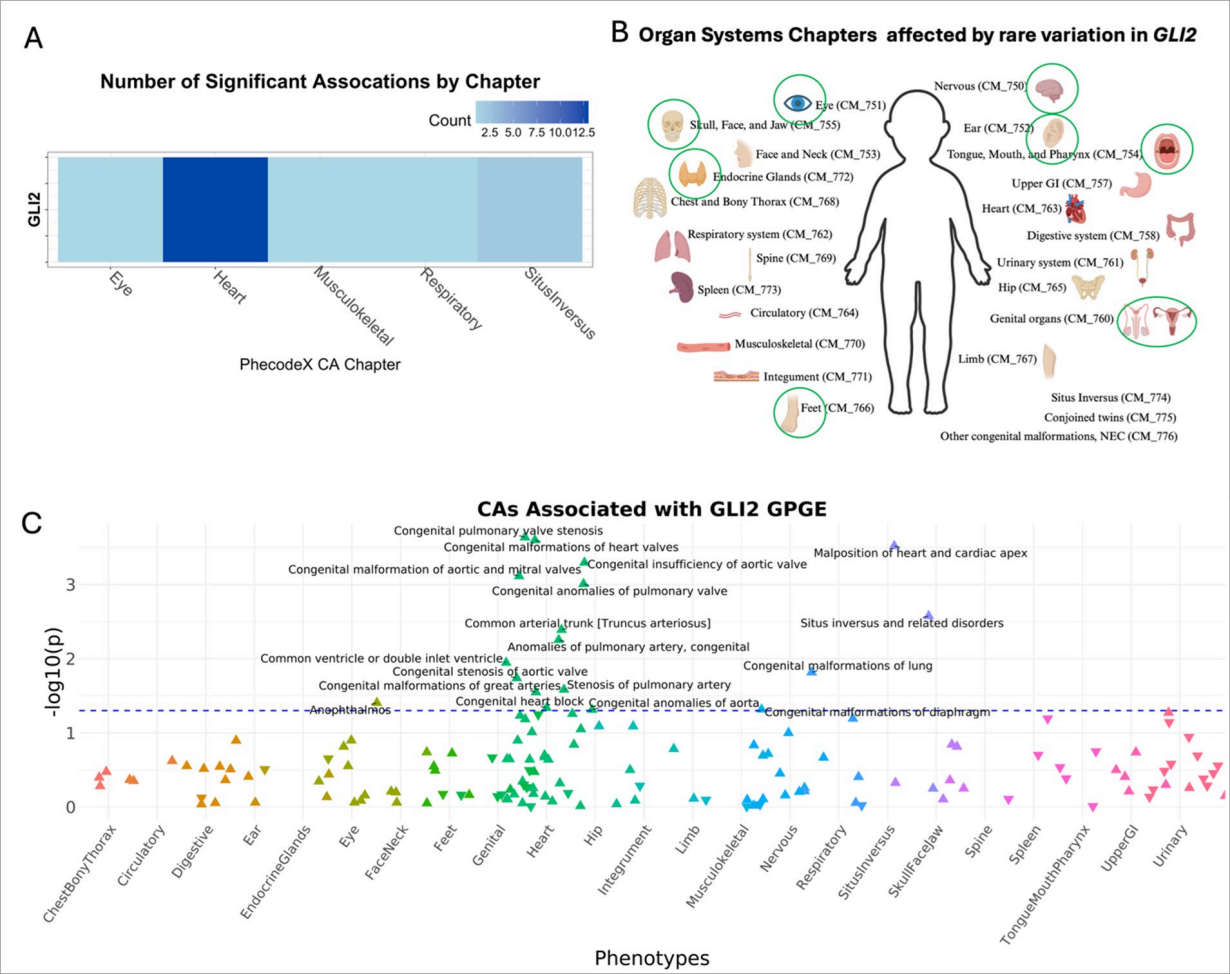
Association study of genetically predicted gene expression (GPGE) of known craniofacial anomaly (CFA) genes and other congenital anomalies (CA). The GPGE of *GLI2* (*165230) is associated with an enrichment of cardiac CAs. The blue dotted line indicates a p-value of 0.05. Direction of the triangle aligns with the direction of the effect.

Because CFAs often co-occur with other CAs, we sought to evaluate whether the curated CFA associated gene list demonstrated more significant associations with other CAs. To do this we tested for the association between the GPGE of 341 CFA-associated genes from the curated list and any CA phecode in both BioVU and eMERGE study populations. Assuming common variation in these genes identified from clinical testing panels for CFAs were specific to craniofacial development, we would expect to find an enrichment of craniofacial phenotypes in the skull face and jaw as well as the mouth, tongue and pharynx phecodeX chapters. While we identified a few significant associations with these phecodes at the least stringent significance threshold (p < 0.05) (Fig. 3A and B), the most significant associations for any of the CFA-associated genes were identified in other organ system CAs. This analysis illustrates that a broad range of phenotypes spanning multiple organ systems are associated with common variation in CFA-associated genes (Fig. 4). Among our gene-based results, we found that the GPGE of *GLI2* (*165230) is associated with 18 different CA phecodes in BioVU (p < 0.05). These include significant associations with CA phecodes from four organ systems, 12 heart, 1 eye, 1 musculoskeletal, and 1 respiratory, as well as 2 situs inversus CA phecodes (Fig. 5, Table 3).

## Discussion

Because most previous genetic studies of CFAs focus on rare variant/ monogenic causes of disease, this work investigated the GPGE of individuals with CFAs and how the GPGE of known CFA genes relate to CAs. We perform a TWAS for individuals with a CFA and a CA-wide PheWAS for the GPGE of genes with a known CFA association. Overall, the results of these analyses suggest that in addition to rare variants, polygenic variation impacting gene expression may contribute to many CAs and may play a role in the penetrance and expressivity of CA syndromes.

As sample sizes for genetic studies have increased, so has our understanding of the complexity of the genetic architecture driving phenotypes such as CFAs through our expanded understanding of how common variation contributes to the diversity of facial morphology and shape.^15,26,27^ Studies of CFAs and polygenic architecture have identified that there are shared genetic features between cleft lip and palate and the size of facial features as well as common variation that affects the penetrance of known cleft clip and palate variants in *PDGFRA* (*173490).^6,7^ Additionally, researchers have proposed that Mendelian CFAs are extreme phenotypes on a continuum of phenotypic variation in facial morphology and that integrating common variation into the study of these phenotypes is essential to understanding their genetic drivers.^27,28^ All of these studies leverage GWAS and polygenic scores to examine common variations contributions to CFAs. However, using an approach that utilizes GPGE allows us to capture the predicted effects of common variant gene expression in a TWAS to identify genes whose altered GPGE is associated with CFA status. By conducting a gene-based analysis of CFAs, we can obtain more biologically interpretable results for our CFA associations.

Out of the 391 genes known to cause CFAs in a monogenic fashion, over 90% are not significantly associated with CFAs in a transcriptome-wide fashion (p < 0.01), highlighting the gap in knowledge when studying CFAs that present in a Mendelian or syndromic fashion. The two genes whose GPGE showed a significant association (p < 0.01) with CFAs in both cohorts, *VAV1* (*164875) and *CYP3A7* (*605340), have not yet been associated with any human disease, but do have known roles in fetal development. *VAV1* (*164875) is a proto-oncogene that is involved with hematopoiesis and T and B cell signaling. The well-established relationship between disrupted oncoprotein signaling, cancer, and congenital anomalies suggests that differential expression of the oncogene *VAV1* (*164875) could be driving the development of some CFAs.^29, 30,24^ Additional research on the differential expression of proto-oncogenes in individuals with congenital anomalies could give additional insight into why those with congenital anomalies are more likely to develop cancer. *CYP3A7* (*605340) encodes a cytochrome P450 “super family” enzyme, *CYP3A7* (*605340), which metabolizes a diverse array of endogenous and exogenous substances, including prescription medications that many pregnant individuals need to maintain their own health such as carbamazepine, diltiazem, caffeine, and nifedipine. *CYP3A7* (*605340) is primarily expressed in fetal liver tissue, being detected as early as 50 days of gestation and decreasing in expression until 24 months of age postnatally.^31,32^ Additionally, *CYP3A7* (*605340) plays a key role in the production of a critical pregnancy hormone, estriol, which has been shown to be an important epigenetic modifier in mice fetuses. Variable expression of *CYP3A7* (*605340) could have dramatic effects on fetal development, and further research can assess the complex interactions of environmental risks and genetic predispositions to CFAs and other CAs.

The observations that only 10% of the previously identified CFA associated genes had a significant GPGE association with CFAs and the strong overlap (21.1%) of individuals with CFAs having other CAs drove us to analyze what CAs were significantly associated with the GPGE of the curated CFA genes. These known CFA genes show more significant GPGE associations with other organ system CAs than the CFAs themselves, suggesting that there are shared genetic and environmental susceptibilities across CAs. The many genetic syndromes that contain multiple CAs support the idea of shared risk factors for many types of CAs. Taken together these results suggest that polygenic variation in CFA-associated genes may relate to developmental changes more broadly and are not necessarily restricted to craniofacial development.

Many genetic syndromes that cause multiple types of CAs demonstrate variable expression and incomplete penetrance, yet the factors causing variable expression/ incomplete penetrance is not well understood. For example, ~ 65% of individuals with 22q11.2 deletion syndrome have a congenital heart defect (CHD) and ~ 67% have a palate abnormality.^33,34^ As another example, we noted that the predicted expression of *GLI2*’s (*165230) is significantly (p < 0.05) associated with many congenital heart defects (CHDs), such as congenital pulmonary valve stenosis (p = 0.0002), congenital malformations of heart valves (p = 0.0002), congenital insufficiency of the aortic valve (p = 0.0005), and ten others (Table 3). *GLI2* (*165230) is classified as a known CFA gene due to its association with two congenital malformation syndromes, Culler-Jones syndrome and Holoprosencephaly 9.^35^ Both syndromes can present with several congenital anomalies, such as cleft lip/palate, microcephaly, polydactyly, but to date CHDs are not associated with either syndrome.^36,37^ Yet *GLI2* (*165230) does have a well-established role in cardiomyogenesis and there is a group of individuals with CHDs that have *GLI2* (*165230) missense variants shown to dysregulate sonic hedgehog signaling, which is crucial for fetal development.^,38,39,40^ The expression of *GLI2* (*165230) in the developing heart suggests CHDs could possibly be a phenotypic expansion for the two *GLI2*-related congenital anomaly syndromes and suggests examining its role in cardiac development. Differences in gene expression could be one factor causing variable expressivity and reduced penetrance that is characteristic of many congenital anomaly syndromes.

One of the main limitations of studying CFAs at biobank scale is that they have a relatively low prevalence and are caused by large-effect rare variants. Both issues affect our statistical power to detect phenotype associations. One way that our analysis attempted to address this limitation is by conducting gene-based analyses and defining our CFA phenotype across multiple phecodes that describe different types of CFAs. Despite using a less stringent p-value threshold, conducting gene-based analyses such as TWAS provides a more interpretable biological unit than single variant analyses. While the analyses in this paper are underpowered, they still provide biologically and clinically meaningful results.^22,23^

Throughout this study we leveraged a set of known CFA genes that were compiled from clinical diagnostic testing for CFAs as well as a GWAS of face shape that was curated and reviewed by a certified genetic counselor. While we tried to make this gene set as comprehensive as possible, we are limited by which genes are currently have a well-established associated with CFAs. The main goal of the work in this study is to try to better understand the genetic drivers of CFAs.

Overall, our results support that both rare and common genetic variants in CFA Mendelian genes may contribute to a variety of CAs and highlights the complexities of the CA phenotypes, suggesting there are shared underlying genetic and environmental risk factors. Further research of CAs through GPGE could help better explain variable presentations and penetrance of CA syndromes.

## Conclusions

TWAS of individuals with CFAs in the BioVU and eMERGE cohorts identified relatively few previously identified CFA-associated genes. The two genes whose GPGE had the strongest association in both cohorts have potential roles in the complex genetic drive of CAs. The predicted expression of genes that have a known Mendelian-association with CFAs are more often significantly associated with other types of CAs in the BioVU and eMERGE cohorts. For example, the predicted expression of *GLI2*, which is associated with a syndrome that can include CFAs, is significantly associated with several CHDs. The results of both analyses suggest that there are overlapping polygenic causes of many types of CAs and that with further research may help explain the variability in how CA syndromes can present.

## Supplementary Material

Supplementary Files

This is a list of supplementary files associated with this preprint. Click to download.

• 17Sept2025SupplementalTables.xlsx

## Figures and Tables

**Table 1:** Demographics of craniofacial cases and controls

	Craniofacial anomalies	Controls	Total
**BioVU (n)**	**635**	**42,810**	**43,445**
EHR-reported sex			
Male	352 (55.4)	17,497 (40.9)	17,849 (41.1%)
Female	283 (44.6)	25,313 (59.1)	25,596 (58.9%)
Age, years	27.1 (23.4)	58.0 (21.1)	57.5 (21.5)
Number of visits	126.0 (135.0)	61.4 (65.5)	62.4 (67.5)
**eMERGE (n)**	**384**	**3,575**	**3,959**
EHR-reported sex			
Male	159 (41.4)	2,014 (56.4)	2,239 (56.6)
Female	225 (58.6)	1,559 (43.6)	1,718 (43.4)
Age, years	17.2 (5.3)	25.2 (7.9)	24.4 (8.1)

**Table 2:** Number of significant results in BioVU and eMERGE craniofacial (CFA) transcriptome-wide association study (TWAS)

Study site	Any gene (p < 0.05)	Any gene (p<0.001)	Known CFA gene (p < 0.05)	Known CFA gene (p < 0.001)
BioVU	1,261	231	11	1
eMERGE	1,260	257	14	4

**Table 3:** Congenital anomaly phecodes associated with genetically predicted expression of *GLI2*

Organ system	CA phecode	Beta	Standard error	p-value
Eye	CM_751.113	0.495	0.240	0.039
Heart	CM_763.212	0.331	0.090	2.291×10^−4^
Heart	CM_763.2	0.145	0.040	2.505 ×10^−4^
Heart	CM_763.232	0.186	0.053	5.033×10^−4^
Heart	CM_763.23	0.158	0.047	7.654 ×10^−4^
Heart	CM_763.21	0.234	0.071	9.777 ×10^−4^
Heart	CM_763.11	0.622	0.216	4.059×10^−3^
Heart	CM_763.15	0.187	0.067	0.006
Heart	CM_763.36	0.259	0.102	0.011
Heart	CM_763.231	0.204	0.086	0.018
Heart	CM_763.152	0.256	0.115	0.026
Heart	CM_763.1	0.095	0.043	0.028
Heart	CM_763.8	0.484	0.242	0.046
Heart	CM_763.14	0.138	0.070	0.049
Musculoskeletal	CM_770.1	0.380	0.192	0.048
Respiratory	CM_762.3	0.377	0.155	0.015
Situs Inversus	CM_774.2	0.672	0.186	3.003 ×10^−4^
Situs Inversus	CM_774	0.451	0.150	0.003

## Data Availability

All data from the primary population at Vanderbilt University Medical Center will be made available by request to the corresponding author pending Institutional approval. Requests for data derived from the secondary population, eMERGE, will be handled per consortium requirements.

## Abbreviations

CFAs: Craniofacial anomalies
CAs: Congenital anomalies
CL/P: Cleft lip and/or palate
HER: Electronic health records
ICD: International Classification of Disease
CM: Congenital malformation
SD: Synthetic derivative
VUMC: Vanderbilt University Medical Center
IRB: Institutional Review Board
PCAs: Principle component analyses
CCHMC: Cincinnati Children’s Hospital Medical Center
GWAS: Genotype-wide association study
TWAS: Transcriptome-wide association study
GPGE: Genetically predicted gene expression
PheWAS: Phenotype-wide association study
CHD: Congenital heart defect
